# Osseous Union of Two Temporary Teeth

**Published:** 1851-10

**Authors:** 


*Osseous Union of Two Temporary Teeth.*—In a note recently received from Dr. Z. M. Kreider of Lancaster, Ohio, is a drawing presenting a labial and lingual view of an inferior central and lateral incisor, with their crowns and roots united. The teeth belonged to first dentition, and were extracted by Dr. H. Scott, dentist, of Lancaster, Ohio, from a German girl, six and a half years of age. Although examples of osseous union of the teeth are not of very frequent occurrence, we have met with several, and there are three, of the temporary teeth, in the museum of the Baltimore College of Dental Surgery, and we have five in our own cabinet. It occurs much more frequently in the temporary than in the permanent teeth. At any rate we have seen some eight or nine examples in the former and only three in the latter. The circumstances connected with the origin and development of the pulps would seem to be more favorable to its occurrence in the one case than in the other, and hence it is, that the teeth of first dentition become more frequently united than those of second.

Dr. Koecker of London, in his "Principles of Dental Surgery," published no longer ago than 1822, expressed his utter disbelief in the occurrence of osseous union of two or more teeth, when, we are assured by Mr. Bell, that at that time, there were several specimens in the museum of Guy's Hospital, which had been placed there some years before, by Mr. Fox. H.

